# Calpain‐2 inhibitor treatment preferentially reduces tumor progression for human colon cancer cells expressing highest levels of this enzyme

**DOI:** 10.1002/cam4.1260

**Published:** 2017-12-06

**Authors:** Michael P. Marciel, Aaron H. Rose, Verena Martinez, David T. Horio, Ann S. Hashimoto, FuKun W. Hoffmann, Pietro Bertino, Peter R. Hoffmann

**Affiliations:** ^1^ Department of Cell and Molecular Biology John A. Burns School of Medicine University of Hawaii Honolulu Hawaii; ^2^ Biotechnology Department University of Applied Sciences Mannheim Mannheim Germany; ^3^ Department of Pathology John A. Burns School of Medicine University of Hawaii Honolulu Hawaii

**Keywords:** Calcium, colorectal cancer, inflammatory bowel disease, intestinal cancer, protease

## Abstract

Calpain‐2 levels are higher in colorectal tumors resistant to chemotherapy and previous work showed calpain‐2 inhibitor therapy reduced inflammation‐driven colorectal cancer, but direct effects of the inhibitor on colon cancer cells themselves were not demonstrated. In the present study, five human colon cancer cell lines were directly treated with a calpain‐2 inhibitor and results showed increased cell death in 4 of 5 cell lines and decreased anchorage‐independent growth for all cell five lines. When tested for levels of calpain‐2, three cell lines exhibited increasing levels of this enzyme: HCT15 (low), HCC2998 (medium), and HCT116 (significantly higher). This was consistent with gel shift assays showing that calpain‐2 inhibitor reduced of NF‐*κ*B nuclear translocation most effectively in HCT116 cells. Ability of calpain‐2 inhibitor to impede tumor progression in vivo was evaluated using intrarectal transplant of luciferase‐expressing cells for these three cell lines. Results showed that calpain‐2 inhibitor therapy reduced tumor growth and increased survival only in mice injected with HCT116 cells. These data suggest calpain‐2 inhibitor treatment may be most effective on colorectal tumors expressing highest levels of calpain‐2.

## Introduction

Calpains are calcium‐dependent cysteine proteases that regulate a wide variety of cellular activities 1. Unlike other proteases, calpains do not degrade target proteins. Calpains cleave their targets between functional domains, thereby altering the signaling or functions of the cleaved domains. There are 15 calpain isoforms, with calpain‐1 and calpain‐2 being the most ubiquitously expressed 2. The activity of these enzymes is regulated not only by calcium binding, but by other factors including subcellular localization, membrane association, and actions of the endogenous inhibitor calpastatin 3. Excessive calpain activity has been implicated in pathogenic processes in many tissues including the gut, brain, eyes, heart, lungs, pancreas, kidneys, vascular system, and skeletal muscle, suggesting that calpain inhibitors may serve as valuable therapeutic agents for a wide variety of diseases 4.

Dysregulated calpain cleavage of target proteins may also drive certain types of cancer. For example, calpain activity has been found to be increased in mesothelioma 5, pancreatic cancer 6, and gastric and colorectal cancer 7. Interest has emerged in inhibitors of calpains as possible therapeutics, but it remains unclear if particular calpains may serve as optimal targets for different cancers or if biomarkers may indicate which patients may benefit most from inhibitor treatment 8. Cancers driven by inflammation, such as colorectal cancer, may rely on calpain enzymes for disease progression 9, 10. The functions of calpain enzymes in colorectal cancer have mainly focused on cell motility due to the role these proteolytic enzymes play in modulating the cytoskeletal machinery. For example, enhanced migration of human HCT116 colon cancer cells through EGF stimulation depended on calpain activity 11. In these same cells, calpain cleavage of focal adhesion kinase (FAK) was crucial for efficient cell motility 9. Similarly, calpain inhibition negatively regulated the actions of Cten, which is a member of the Tensin that induces cell motility 12. These studies relied on pan calpain inhibitors that maximize inhibitory effects, but this may not be optimal for antitumor therapy. For example, a study in rats found that intact calpain‐9 activity may be important for drug‐induced apoptosis of colorectal cancer cells 13. In fact, calpain activity has been shown to actually promote cell survival through *β*‐catenin/Wnt signaling during development 14, and cancers may exploit these antiapoptotic pathways for survival as well. Thus, a better understanding of how inhibition of particular calpain family members may interfere with colorectal cancer cell progression is needed for better development of therapies.

Studies in our laboratory have focused on calpain‐2 mainly due to its role in promoting the activation of macrophages and inflammation derived from secretion of cytokines, and the important role for increased calpastatin in limiting calpain‐2 during inflammatory bowel disease 15, 16. This led our laboratory to investigate the effects of calpain‐2 inhibitor therapy using a mouse model of colitis and associated colorectal cancer induced by azoxymethane and dextran sulfate sodium. Using a small molecule inhibitor of calpain‐2, we found that both colitis and colorectal cancer were reduced with calpain‐2 inhibitor treatment 17. This effect was mediated through the inhibition of calpain‐2 cleavage of I*κ*B that in turn reduced nuclear translocation of NF‐*κ*B. However, it was not clear whether the reduction in colorectal cancer was due mainly to lower inflammation required for growth of the tumors or if there were direct antigrowth effects on the colon tumors themselves. Interestingly, high levels of calpain‐2 have been shown to play a role in chemotherapeutic‐resistant colorectal cancer through mechanisms involving NF‐*κ*B activation 18. In order to determine how calpain‐2 inhibition directly affects human colon cancer cells, we carried out experiments described herein using NCI‐60 validated human colon cancer cell lines to test the effects of calpain‐2 inhibition on cell death, colony formation, and in vivo tumor progression. In vitro and In vivo results showed that the effects found on different colon cancer cell lines corresponded to calpain‐2 levels in these cells.

## Materials and Methods

### Mice, cells, and reagents

Male athymic nude (*nu/nu*) mice were purchased from the Jackson Laboratory (Bar Harbor, ME) and housed in microisolator cages under specific pathogen‐free conditions. At 8 weeks of age, mice were used for experiments and all animal protocols were approved by the University of Hawaii Institutional Animal Care and Use Committee. NCI‐60 cell lines were obtained from the University of Hawaii Cancer Center that included four human mesothelioma cell lines (HMESO, ROB, Mill, and REN) and five human colorectal cancer cell lines (HCC2998, COLO205, HCT116, HT29, HCT15, and KM12). Cells were tested for mycoplasma using PCR (Sigma) and used in early passage (<10). Cells were cultured in RPMI media with 10% fetal bovine serum and 1% antibiotic–antimycotic solution (all from Gibco/Thermo Fisher Scientific). For in vivo experiments, HCC2998, HCT116, and HCT15 stable cell lines expressing luciferase were generated using a piggyBac plasmid as described previously 19. The calpain‐2 inhibitor, zLLY‐CH2F, was purchased from EMD Millipore (Life Science) and used at 20 *μ*g/mL final concentration in cell culture experiments and 0.75 mg/kg in mice as described previously 17. Propidium iodide was purchased from Thermo Fisher and stock solutions were made at 1 mg/mL in water. Antibodies used included anti‐calpain‐2 and anti‐GAPDH (Santa Cruz Biotechnology, Santa Cruz, CA), anti‐calpastatin (Cell Signaling, Danvers, MA), and anti‐calpain‐1 (Millipore, Billerica, MA). Raji nuclear extracts (Active Motif, Inc., Carlsbad, CA) were used as a positive control in electrophoretic mobility shift assays (EMSA). Agar for colony formation assays was purchased from VWR. A calpain activity assay kit (Abcam, Cambridge, MA) was used as described previously 15.

### In vivo colorectal cancer assay

An intrarectal injection model was carried out for colon cancer as described previously 20. For these experiments, we harvested each stable luciferase‐transfected cell line at ~70% confluency and generated cell suspensions (10^6^ cells in 100 *μ*L PBS) that were injected into the submucosa of protracted rectum of anesthetized mice. This was carried out with the use of a dissection microscope and a small tube inserted into the rectum to provide an opening and allow visualization of the injection site. Mice were intraperitoneally (i.p.) injected daily with either vehicle control (DMSO) or 0.75 mg/kg calpain inhibitor, which was previously shown to be the optimal dosage that inhibited calpain‐2 in colons of mice 17. To assess tumors containing luciferase‐positive cells in vivo, bioluminescent signals from luciferase‐positive tumor cells were monitored using the IVIS Lumina (Perkin Elmer, Waltham, MA, USA). Luciferase signals were quantified as total photon counts using Living ImageTM software Hopkinton, MA. Mice were monitored for morbidity indicated by extreme fatigue, poor grooming, or strained motility. In separate experiments, the same transplantation and inhibitor therapy protocols were followed, but instead of conducting luciferase activity measurements the mice were sacrificed at 4 weeks of age, perianal tissues excised, and histochemical analyses of hematoxylin and eosin (H&E) staining. Examination of H&E slides were performed by a pathologist. When poor health reached significant levels due to tumor growth, mice were euthanized and time spent on protocol recorded for survival analyses.

### Soft agar assays

Human colon cancer cell lines were cultured in agar for 21 days and the media was changed biweekly with either 1 mL of culture media containing either calpain‐2 inhibitor (20 *μ*g/mL) or the same volume of vehicle (DMSO). The soft agar consisted of two different layers, each of which contained 1% agar. The agar was boiled and cooled down by adding an equal volume of 2X RPMI containing 20% of FBS to the agar to give a final concentration of 0.5% agar, 1X RPMI, and 10% FBS. A total volume of 1.5 mL of the mixture was added to each well of a 6‐well plate and was set aside for 30 min to solidify. During solidification, the top agar layer was prepared by repeating the same procedure as above. Before adding the mixture to the wells, a total number of 10^4^ cells per well were added and mixed gently. The agar containing cells were added to the top of the base layer and allowed to solidify for another 30 min. Cells were fed with 1 mL of either RPMI containing DMSO vehicle or 20 *μ*g/mL of calpain‐2 inhibitor. A change of medium was performed twice per week for a period of 21 days. Colonies were stained by adding a total amount of 500 *μ*L of 0.005% crystal violet for 1 h. After incubation, the staining solution was removed and the stained colonies were counted.

### Cell death assays

Cells were seeded in quadruplicate at 10^4^ cells per well in a sterile 96‐well plate for each condition and then allowed to recover for 18 h. Cells were either treated with 20 *μ*g/mL calpain‐2 inhibitor or an equal volume of vehicle DMSO as a control for 14 h. Cells were spun down at 300*g* for 3 min and media removed, the cells were washed once with 200 *μ*L PBS and resuspended in PBS. From a stock solution of propidium iodide (1.0 mg/mL) a working solution (0.1 mg/mL) was added at 1:100 for a final concentration of 1 *μ*g/mL. Cells were then transferred to flow cytometry tubes and analyzed by a FACScaliber (BD Biosciences) and data analyzed using FlowJo software Ashland, OR for propidium iodide‐positive cells.

### Western blots and EMSA

Western blotting methods were performed as described previously 21, and bands were visualized using a Li‐Cor Odyssey Lincoln, NE. Densitometry was used to analyze band intensity using ImageJ. The EMSA were performed on cell nuclear lysates using oligonucleotides end labeled with IR700 dye (Licor, Inc., Lincoln, NE) as described previously 17.

### Statistical analyses

Comparison of means for two groups (e.g., control vs. inhibitor) was carried out using an unpaired Student's *t* test using GraphPad Prism version 4.0. In assays involving three or more groups (e.g., three cell lines) a one‐way ANOVA was used to with Tukey's posttest used to compare means of each group. All comparisons were considered significant at *P* < 0.05. GraphPad Prism was also used to generate Kaplan–Meier survival curves and data analyzed with a log‐rank Mantel–Cox test with significance considered at *P* < 0.05.

## Results

### Calpain‐2 inhibitor exhibits biostatic effects on colon cancer cells

**Figure 1:**
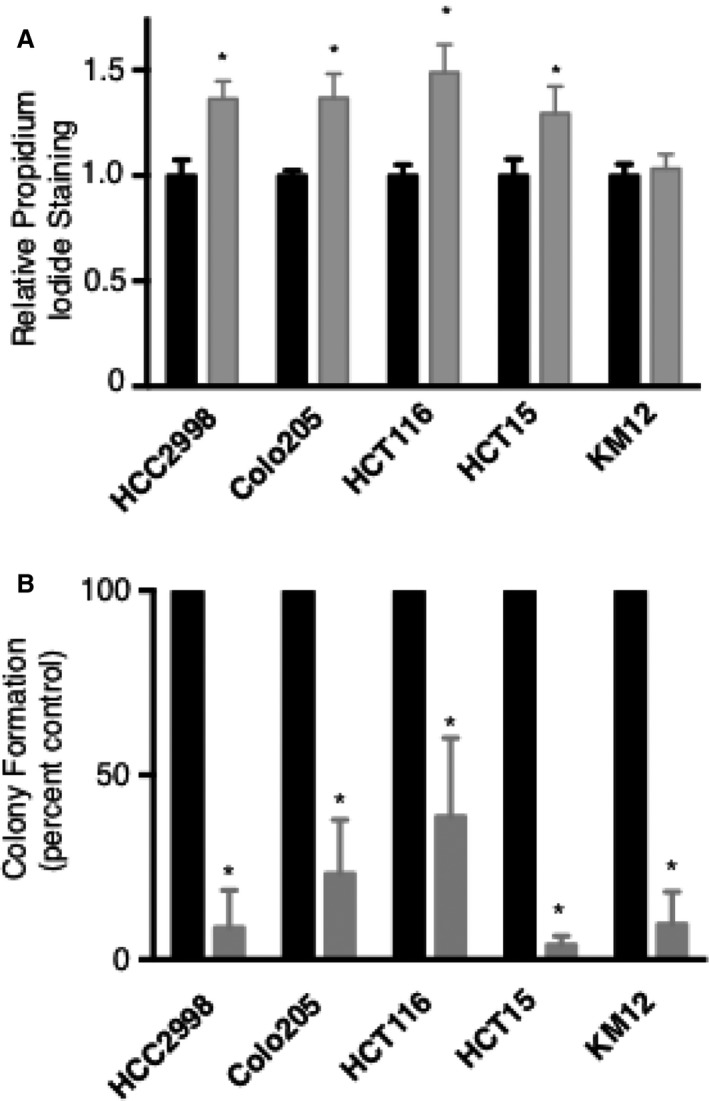
Calpain‐2 inhibitor treatment has cytotoxic and biostatic effects on human colon cancer cell lines. Five NCI‐60 human colon cancer cell lines were treated with either vehicle control (DMSO) or calpain‐2 inhibitor (20 *μ*g/mL). Cell permeability was evaluated by propidium iodide (PI) staining using flow cytometry, with PI positivity for inhibitor compared to control for each cell line. (B) Soft agar colony formation assays were performed to evaluate anchorage‐independent growth. Colonies were enumerated and inhibitor treatment compared to controls for each cell line. Results are expressed as mean ± SEM (*N* = 4) with *P <* *0.05.

Calpain inhibitor studies have mainly used pan calpain inhibitors and focused on migration scratch assays. However, we were interested in specifically inhibiting calpain‐2 and a more comprehensive analyses of its cytotoxic and biostatic effects on colon cancer cells. Thus, we evaluated a panel of NCI‐60 human colon cancer cell lines treated with vehicle control (DMSO) or calpain‐2 inhibitor using propidium iodide staining and soft agar colony assays. The cell lines tested included HCC2998, Colo205, HCT116, HCT15, and KM12 colon cancer cell lines and the dose of calpain‐2 was used shown previously to be effective and specific 15, 17. We found that 4 of 5 cell lines exhibited increased cell death compared to controls as indicated by propidium iodide staining (Fig. 1A). In colony formation assays, all of the five cell lines tested showed significantly reduced colony growth with calpain‐2 inhibitor treatment (Fig. 1B). Together, these data suggest that calpain‐2 inhibitor treatment induces cytotoxicity in most human colorectal cancer cell lines and was effective in inhibiting anchorage‐independent growth of cells for all cell lines tested.

### Calpain‐2 levels vary in different cancer cell lines

**Figure 2:**
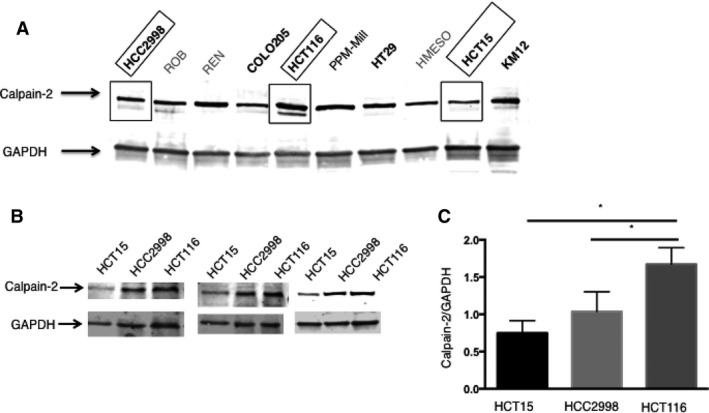
Levels of calpain‐2 vary in different cancer cells. (A) Western blot was used to evaluate calpain‐2 levels in NCI‐60 cell lines including five colon cancer cell lines (bolded) and, for comparison, four mesothelioma cell lines. Three colon cancer cell lines highlighted by boxes showed increasing levels of calpain‐2: HCT15 (low), HCC2998 (medium), and HCT116 (high). (B) Three independent western blots were carried out showing that these cell lines had increasing levels of calpain‐2. (C) Densitometry was performed on band intensity and results confirmed highest levels in HCT116. Results are expressed as mean ± SEM (*N* = 3) with *P <* *0.05.

We tested lysates from the five colon cancer cell lines described above for levels of calpain‐2. For comparison, we included four NCI‐60 mesothelioma cell lysates in the western blot analysis. Results indicated that calpain‐2 is expressed in all cell lines with some variation in levels (Fig. 2A). We also examined total calpain activity as well as levels of calpain‐1 and calpastatin (Figs. S1 and S2). Since calpain‐2 was most consistently expressed and exhibited interesting patterns of increasing levels in certain colon cancer cell lines, we next focused on three colon cancer cell lines that showed different levels of calpain‐2: HCT15 (low), HCC2998 (medium), and HCT116 (high). Three independent western blots were carried out showing that these cell lines had increasing levels of calpain‐2, with HCT116 cells exhibiting significantly higher levels (Fig. 2B and C).

**Figure 3:**
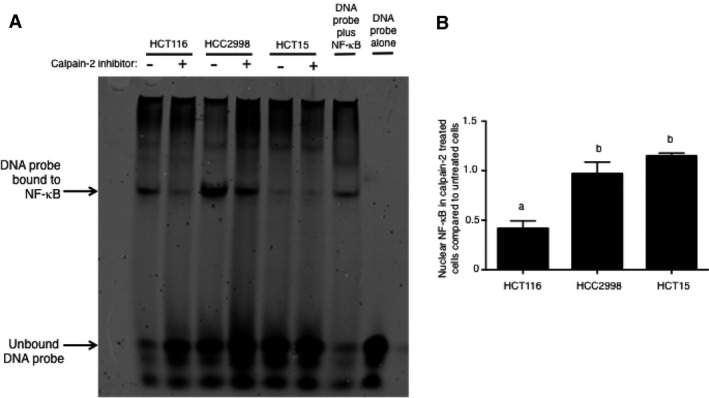
Calpain‐2 inhibitor treatment shows a strong inhibitory effect on NF‐*κ*B nuclear translocation in HCT116 colon cancer cells. (A) A representative image of EMSA performed on nuclear lysates prepared from HCT116, HCC2998, and HCT15 cells that were treated with calpain‐2 inhibitor (20 *μ*g/mL) or equal volume of vehicle control (DMSO). Controls included probe only to indicate region where unbound probe is located and nuclear lysate from Raji cells incubated with probe to indicate region where probe bound to NF‐*κ*B was located. Results indicated that HCT116 showed decreased bound probe in the nucleus with calpain‐2 inhibitor treatment. (B) Densitometry from three repeats showed that the ratio of bound to unbound probe in calpain‐2‐treated compared to untreated cells was significantly lower in the nuclear lysates from HCT116 cells. Results represent three independent experiments and a one‐way ANOVA was used to analyze groups with Tukey's posttest used to compare means of each group. Results are expressed as mean ± SEM and means without a common letter differ, *P* < 0.05.

It has been previously shown that inhibition of calpain‐2 can decrease I*κ*B degradation leading to less NF‐*κ*B translocation to the nucleus in some cells and this has implications in colorectal cancer 17, 18. To evaluate if HCT15, HCC2998, and HCT116 colon cancer cell lines responded differently to calpain‐2 inhibitor treatment in terms of NF‐*κ*B nuclear localization, we carried out an EMSA. As shown in Figure 3, HCT116 showed the strongest response to calpain‐2 inhibitor treatment. This was evident by the presence of nuclear NF‐*κ*B bound to probe in untreated cells that was greatly reduced by calpain‐2 inhibitor treatment. Nuclear NF‐*κ*B bound to probe in HCC2998 cells was slightly reduced by calpain‐2 inhibition, but there still remained large amounts of nuclear NF‐*κ*B. HCT15 cells did not have large amounts of detectable nuclear NF‐*κ*B and inhibitor treatment did not affect these levels. The inhibitor treatment experiments followed by EMSA were repeated (*N* = 3) and densitometry of bound to unbound ratios was performed, showing that HT116 cells consistently exhibited the strongest response to calpain‐2 inhibitor treatment. These results correlate with those found for each cell line described above in that HCT116 has the highest levels of calpain‐2 and is most sensitive to the inhibitor of this enzyme.

### Calpain‐2 inhibitor inhibits HCT116 tumor growth and increases survival

**Figure 4:**
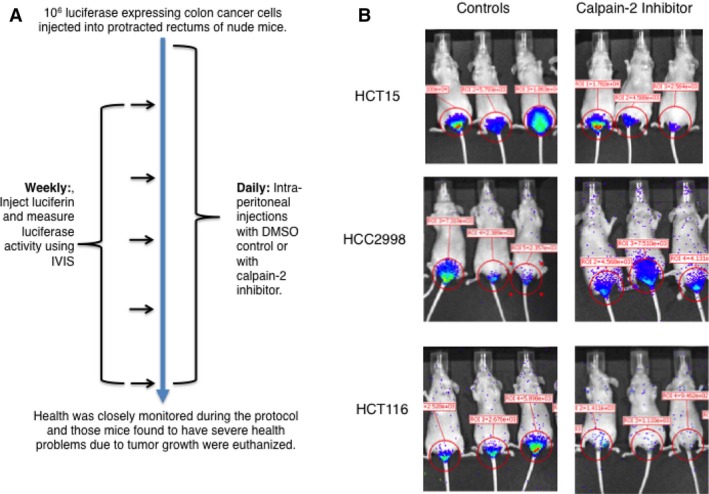
An intrarectal injection model of colorectal cancer growth in nude mice demonstrates effective reduction of HCT116 tumors with calpain‐2 treatment. (A) As described in the Materials and Methods section, male nude mice were injected with 10^6^ cells stably transfected with luciferase into the submucosa of protracted rectums and treated daily with either vehicle control (DMSO) or calpain‐2 inhibitor (0.75 mg/kg). (B) An IVIS instrument was used to detect luciferase activity in anesthetized mice, and representative images are shown for three mice of each group at the 4‐week time point.

**Figure 5:**
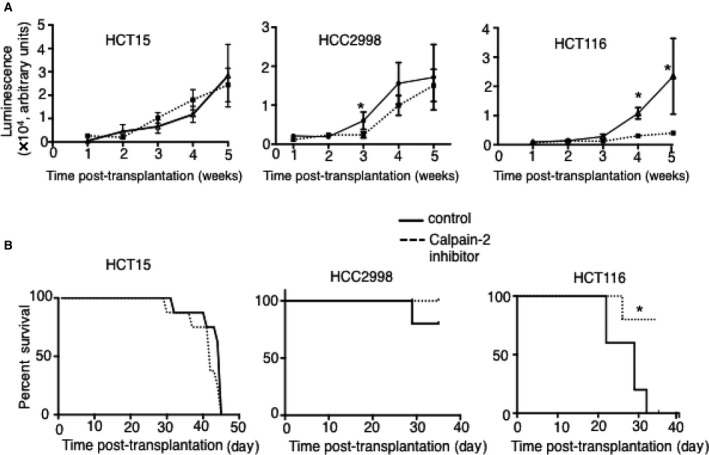
HCT116 tumor growth is hindered by calpain‐2 inhibitor treatment. (A) Three different colon cancer cell lines transplanted into submucosa of rectums grew into tumors over a 5‐week period. Calpain‐2 inhibitor treatment effectively reduced the growth of HCT116, but had limited effects on HCC2998 and no effect on HCT15 growth. Results are expressed as mean ± SEM (*N* = 5 per group) with *P <* *0.05. (B) Survival analyses showed significant effects of calpain‐2 inhibitor therapy for mice injected with HCT116 cells, but not with HCT15 or HCC2998 cell lines.

To determine if in vivo growth of the three colon cancer cell lines described above was affected by calpain‐2 inhibitor therapy, we utilized an intrarectal transplant model described previously 20. In brief, 10^6^ cells stably transfected with luciferase were injected into the submucosa of protracted rectums in nude (*nu/nu*) mice. Mice were then treated daily with either vehicle control (DMSO) or calpain‐2 inhibitor at a previously optimized dose 17, and in vivo luciferase activity was measured weekly to monitor tumor growth (Fig. 4). The luciferase images suggest that the tumors injected into submucosal of distal colons spread by invading adjacent tissues, and this was confirmed by histological examination of H&E‐stained tissues (Fig. S3). In the vehicle control‐treated mice, all three cell lines produced tumors that progressed over a 5‐week period, but calpain‐2 inhibitor therapy effectively inhibited tumor growth only for HCT116 cells (Fig. 5A). The mice injected with HCC2998 cells had a slight reduction in tumor growth with calpain‐2 inhibitor treatment during weeks 3 and 4, but no significant differences were detected by week 5. Consistent with these results, calpain‐2 inhibitor increased survival only in the mice injected with HCT116 cells (Fig. 5B).

## Discussion

The findings presented herein show a correlation between levels of calpain‐2 and effectiveness of calpain‐2 therapy for treating colon cancer. With the development of precision medicine, it is likely that information gathered on patient biopsy or resection samples will need to be utilized for guiding decisions on how responsive colon cancer cases will be to calpain‐2 inhibitors. This is highlighted by studies showing that expression of calpain‐1 and ‐2 along with calpastatin can vary within cohorts of cancer patients and these data may be used to predict disease progression or survival 22, 23. While calpain‐1 and calpastatin levels varied widely in the cancer cell lines we examined in this study, calpain‐2 levels were consistently detected and showed interesting patterns of low, moderate, and high expression. For human samples, new techniques have been developed to accurately analyze calpain‐2 levels 24, which may be utilized if calpain‐2 inhibitor therapy becomes approved for colorectal cancer patients. In fact, there is an emerging interest in better developing the discipline of “degradomics” for assessing proteases/substrates along with breakdown products as biomarkers for cancer and other diseases 25. Calpains have been suggested to be important elements of degradomics 24, and our data provide support for this notion by showing how calpain‐2 levels may predict response to calpain‐2 inhibitor therapy.

Our data showing increased calpain‐2 in HCT116 cells is important given the interesting data from previous studies suggesting that calpain‐2 is important for drug resistance in colorectal cancer. In particular, CPT‐11 (irinotecan) is the first‐line chemotherapy for advanced stage colorectal cancer and many patients develop resistance after several courses of treatment 26. The causative factors involved in this resistance are complex, use of xenograft of HT‐29 human colon cancer cells in nude mice treated with this chemotherapeutic led to resistant tumors (HT‐29) that had upregulated calpain‐2 18. Moreover, upregulated calpain‐2 was directly related to overactive, direct cleavage of I*κ*B leading to increased NF‐*κ*B in the HT‐29R cells. The authors of this study suggested that calpain 2‐dependent I*κ*B degradation mediates CPT‐11 secondary resistance in colorectal cancer xenografts. Thus, targeted therapies directed against calpain 2 may represent a novel strategy to enhance the anticancer efficacy of CPT‐11 chemotherapy. Our data suggest that this may be true since only those colon cancer cell expressing high levels of calpain‐2 will be impacted by calpain‐2 inhibitor therapy.

In order for higher levels of calpain‐2 to exert protumor effects, there is a need for this enzyme to bind to calcium within the cell in order to become activated. During transformation of healthy cells to colorectal cancer cells, calcium homeostasis and signaling are altered in a process referred to as calcium remodeling 27. In fact, several different types of cancer demonstrate altered intracellular calcium homeostasis that is involved in tumor initiation, angiogenesis, progression, and metastasis 28. This has led to an emerging effort to develop calcium chelators, inhibitors or regulators for calcium channels/transporters, or modulators of calcium‐ATPase pumps as anticancer drugs. However, it has been noted that there is heterogeneity in calcium handling machinery in different types of cancers 29, and this may limit a broad application of chemotherapeutics that target store‐operated calcium entry in cancers 30. Our data along with others suggest that calpain‐2 may be an effective target for some cases of colorectal cancer 17, 18. Screening tumor biopsy samples or surgical resections for levels of calpain‐2 is a novel approach for adding a level of precision medicine to the treatment of colon cancer. The results of the present findings suggest that levels of calpain‐2 may serve as an effective biomarker for predicting successful cancer‐static effects of calpain‐2 inhibitor therapy.

## Conflict of Interest

None declared.

## Supporting information


**Figure S1**. Total calpain activity normalized to total protein was measured for the different cell lines (10^7^ cells) and graphed from highest to lowest.

**Figure S2**. Western blot analyses of calpain‐1, calpain‐2, and calpastatin were carried out for colon cancer cell lines (red) and mesothelioma cell lines. Calpain‐2 was found to be most consistently expressed, and interesting patterns were apparent such as cell lines expressing low (Hct15), moderate (HCC2998), and high (HCT116) levels of this enzyme.

**Figure S3**. Tissues from perianal area of mice were excised and 10% formalin‐fixed tissues sectioned and stained with hematoxylin and eosin (H&E).

## References

[1] Saido, T. C. , H. Sorimachi , and K. Suzuki . 1994 Calpain: new perspectives in molecular diversity and physiological‐pathological involvement. FASEB J. 8:814–822. 8070630

[2] Sorimachi, H. , S. Hata , and Y. Ono . 2011 Impact of genetic insights into calpain biology. J. Biochem. 150:23–37. 21610046 10.1093/jb/mvr070

[3] Perrin, B. J. , and A. Huttenlocher . 2002 Calpain. Int. J. Biochem. Cell Biol. 34:722–725. 11950589 10.1016/s1357-2725(02)00009-2

[4] Potz, B. A. , M. R. Abid , and F. W. Sellke . 2016 Role of calpain in pathogenesis of human disease processes. J. Nat. Sci. 2:pii: e218. 27747292 PMC5065022

[5] Tabata, C. , R. Tabata , and T. Nakano . 2016 Calpeptin prevents malignant pleural mesothelioma cell proliferation via the Angiopoietin1/Tie2 System. Asian Pac. J. Cancer Prev. 17:3405–3409. 27509983

[6] Yoshida, M. , Y. Miyasaka , K. Ohuchida , T. Okumura , B. Zheng , N. Torata , et al. 2016 Calpain inhibitor calpeptin suppresses pancreatic cancer by disrupting cancer‐stromal interactions in a mouse xenograft model. Cancer Sci. 107:1443–1452. 27487486 10.1111/cas.13024 PMC5084662

[7] Ivanova, E. V. , I. V. Kondakova , L. V. Spirina , S. G. Afanas'ev , A. V. Avgustinovich , and O. V. Cheremisina . 2014 Chymotrypsin‐like activity of proteasomes and total calpain activity in gastric and colorectal cancer. Bull. Exp. Biol. Med. 157:781–784. 25342484 10.1007/s10517-014-2666-y

[8] Moretti, D. , B. Del Bello , G. Allavena , and E. Maellaro . 2014 Calpains and cancer: friends or enemies? Arch. Biochem. Biophys. 564:26–36. 25305531 10.1016/j.abb.2014.09.018

[9] Sundaramoorthy, P. , J. J. Sim , Y. S. Jang , S. K. Mishra , K. Y. Jeong , P. Mander , et al. 2015 Modulation of intracellular calcium levels by calcium lactate affects colon cancer cell motility through calcium‐dependent calpain. PLoS ONE 10:e0116984. 25629974 10.1371/journal.pone.0116984 PMC4309579

[10] Selvakumar, P. , E. Smith‐Windsor , K. Bonham , and R. K. Sharma . 2006 N‐myristoyltransferase 2 expression in human colon cancer: cross‐talk between the calpain and caspase system. FEBS Lett. 580:2021–2026. 16530191 10.1016/j.febslet.2006.02.076

[11] Gueguinou, M. , T. Harnois , D. Crottes , A. Uguen , N. Deliot , A. Gambade , et al. 2016 SK3/TRPC1/Orai1 complex regulates SOCE‐dependent colon cancer cell migration: a novel opportunity to modulate anti‐EGFR mAb action by the alkyl‐lipid Ohmline. Oncotarget 7:36168–36184. 27102434 10.18632/oncotarget.8786 PMC5094991

[12] Thorpe, H. , M. Akhlaq , D. Jackson , S. Al Ghamdi , S. Storr , S. Martin , et al. 2015 Multiple pathways regulate Cten in colorectal cancer without a Tensin switch. Int. J. Exp. Pathol. 96:362–369. 26852686 10.1111/iep.12154 PMC4744826

[13] Saini, M. K. , and S. N. Sanyal . 2014 Piroxicam and c‐phycocyanin prevent colon carcinogenesis by inhibition of membrane fluidity and canonical Wnt/beta‐catenin signaling while up‐regulating ligand dependent transcription factor PPARgamma. Biomed. Pharmacother. 68:537–550. 24721324 10.1016/j.biopha.2014.03.007

[14] Konze, S. A. , L. van Diepen , A. Schroder , R. Olmer , H. Moller , A. Pich , et al. 2014 Cleavage of E‐cadherin and beta‐catenin by calpain affects Wnt signaling and spheroid formation in suspension cultures of human pluripotent stem cells. Mol. Cell Proteomics 13:990–1007. 24482122 10.1074/mcp.M113.033423 PMC3977196

[15] Huang, Z. , F. W. Hoffmann , R. L. Norton , A. C. Hashimoto , and P. R. Hoffmann . 2011 Selenoprotein K is a novel target of m‐calpain, and cleavage is regulated by Toll‐like receptor‐induced calpastatin in macrophages. J. Biol. Chem. 286:34830–34838. 21849499 10.1074/jbc.M111.265520 PMC3186414

[16] Huang, Z. , A. H. Rose , F. W. Hoffmann , A. S. Hashimoto , P. Bertino , T. Denk , et al. 2013 Calpastatin prevents NF‐kappaB‐mediated hyperactivation of macrophages and attenuates colitis. J. Immunol. 191:3778–3788. 23986533 10.4049/jimmunol.1300972 PMC3783572

[17] Rose, A. H. , Z. Huang , C. Mafnas , J. H. Hara , F. W. Hoffmann , A. S. Hashimoto , et al. 2015 Calpain‐2 Inhibitor therapy reduces murine colitis and colitis‐associated cancer. Inflamm. Bowel Dis. 21:2005–2015. 26076056 10.1097/MIB.0000000000000471 PMC4540674

[18] Fenouille, N. , S. Grosso , S. Yunchao , D. Mary , R. Pontier‐Bres , V. Imbert , et al. 2012 Calpain 2‐dependent IkappaBalpha degradation mediates CPT‐11 secondary resistance in colorectal cancer xenografts. J. Pathol. 227:118–129. 22069124 10.1002/path.3034

[19] Bertino, P. , J. Urschitz , F. W. Hoffmann , B. R. You , A. H. Rose , W. H. Park , et al. 2014 Vaccination with a piggyBac plasmid with transgene integration potential leads to sustained antigen expression and CD8(+) T cell responses. Vaccine 32:1670–1677. 24513010 10.1016/j.vaccine.2014.01.063 PMC3973154

[20] Kashtan, H. , M. Rabau , J. B. Mullen , A. H. Wong , J. C. Roder , B. Shpitz , et al. 1992 Intra‐rectal injection of tumour cells: a novel animal model of rectal cancer. Surg. Oncol. 1:251–256. 1341258 10.1016/0960-7404(92)90072-s

[21] Meiler, S. , Y. Baumer , Z. Huang , F. W. Hoffmann , G. J. Fredericks , A. H. Rose , et al. 2013 Selenoprotein K is required for palmitoylation of CD36 in macrophages: implications in foam cell formation and atherogenesis. J. Leukoc. Biol. 93:771–780. 23444136 10.1189/jlb.1212647 PMC3629442

[22] Storr, S. J. , S. Zhang , T. Perren , M. Lansdown , H. Fatayer , N. Sharma , et al. 2016 The calpain system is associated with survival of breast cancer patients with large but operable inflammatory and non‐inflammatory tumours treated with neoadjuvant chemotherapy. Oncotarget 7:47927–47937. 27323818 10.18632/oncotarget.10066 PMC5216989

[23] Luo, W. , Z. Ren , S. Gao , H. Jin , G. Zhang , L. Zhou , et al. 2016 Clinical correlation of calpain‐1 and glypican‐3 expression with gallbladder carcinoma. Oncol. Lett. 11:1345–1352. 26893741 10.3892/ol.2016.4079 PMC4734278

[24] El‐Assaad, A. , Z. Dawy , G. Nemer , and F. Kobeissy . 2017 Novel bioinformatics‐based approach for proteomic biomarkers prediction of Calpain‐2 & Caspase‐3 protease fragmentation: application to betaII‐Spectrin protein. Sci. Rep. 7:41039. 28112201 10.1038/srep41039 PMC5253643

[25] Doucet, A. , G. S. Butler , D. Rodriguez , A. Prudova , and C. M. Overall . 2008 Metadegradomics: toward in vivo quantitative degradomics of proteolytic post‐translational modifications of the cancer proteome. Mol. Cell Proteomics 7:1925–1951. 18596063 10.1074/mcp.R800012-MCP200

[26] Siegel, R. , C. Desantis , and A. Jemal . 2014 Colorectal cancer statistics. CA Cancer J. Clin. 64:104–117. 24639052 10.3322/caac.21220

[27] Villalobos, C. , D. Sobradillo , M. Hernandez‐Morales , and L. Nunez . 2017 Calcium remodeling in colorectal cancer. Biochim. Biophys. Acta 1864:843–849. 10.1016/j.bbamcr.2017.01.005 28087343

[28] Xie, J. , H. Pan , J. Yao , Y. Zhou , and W. Han . 2016 SOCE and cancer: recent progress and new perspectives. Int. J. Cancer 138:2067–2077. 26355642 10.1002/ijc.29840 PMC4764496

[29] Hooper, R. , M. R. Zaidi , and J. Soboloff . 2016 The heterogeneity of store‐operated calcium entry in melanoma. Sci. China Life Sci. 59:764–769. 27417567 10.1007/s11427-016-5087-5 PMC4991353

[30] Cui, C. , R. Merritt , L. Fu , and Z. Pan . 2017 Targeting calcium signaling in cancer therapy. Acta Pharm. Sin B. 7:3–17. 28119804 10.1016/j.apsb.2016.11.001 PMC5237760

