# Supplementary figures and images for: Calpain‐2 inhibitor treatment preferentially reduces tumor progression for human colon cancer cells expressing highest levels of this enzyme
Source: Cancer Med. 2017 Dec 6;7(1):175–83. doi: 10.1002/cam4.1260 (PMC5773958; doi:10.1002/cam4.1260)

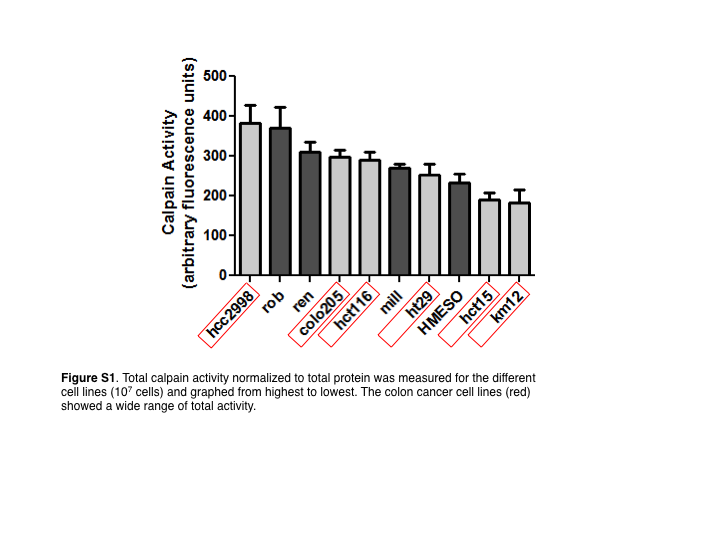

Supplement: Supplementary file 1 — Figure S1. Total calpain activity normalized to total protein was measured for the different cell lines (107 cells) and graphed from highest to lowest. [file CAM4-7-175-s001.tiff]

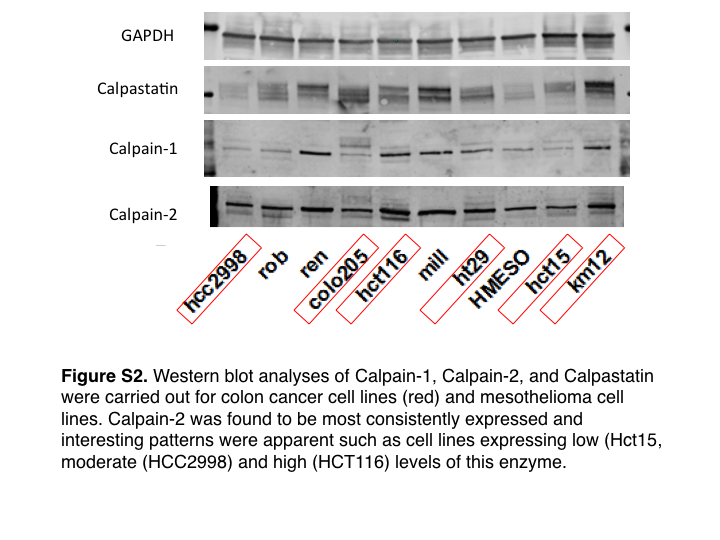

Supplement: Supplementary file 2 — Figure S2. Western blot analyses of calpain‐1, calpain‐2, and calpastatin were carried out for colon cancer cell lines (red) and mesothelioma cell lines. Calpain‐2 was found to be most consistently expressed, and interesting patterns were apparent such as cell lines expressing low (Hct15), moderate (HCC2998), and high (HCT116) levels of this enzyme. [file CAM4-7-175-s002.tiff]

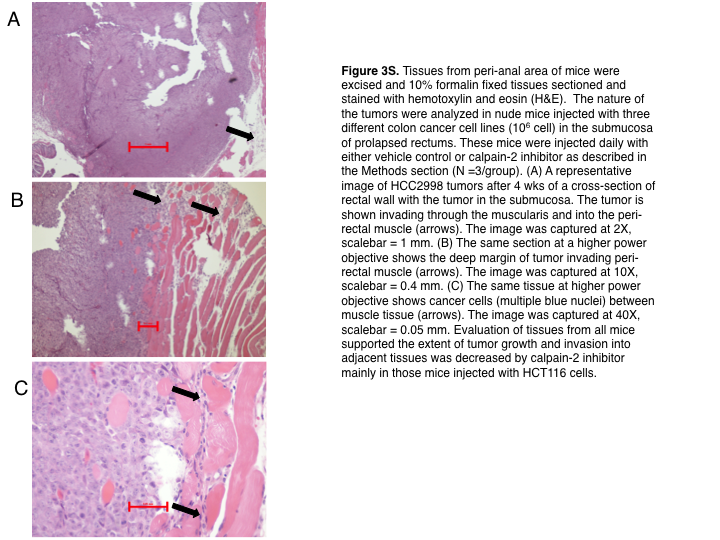

Supplement: Supplementary file 3 — Figure S3. Tissues from perianal area of mice were excised and 10% formalin‐fixed tissues sectioned and stained with hematoxylin and eosin (H&E). [file CAM4-7-175-s003.tiff]
